# Implantation of Teeth

**Published:** 1886-11

**Authors:** 


### IMPLANTATION OF TEETH.

Some months since we received from the author, Wm. J. Younger, M. D., of San Francisco, Cal., a pamphlet with the title "Transplantation of Teeth into Artificial Sockets," in which was recounted his experience in forming artificial sockets in jaws which had long lost their natural teeth, and the transplanting of extracted teeth to them. We were aware that sound teeth had often enough been placed in the sockets from which diseased ones had been freshly removed, but this drilling of holes in edentulous jaws and inserting teeth seemed so unscientific, unphysiological and non-surgical, that we were rather amazed at the assurance of the dentist who had calmly proposed such a thing. We were taught that the retention of a tooth in the jaw depended upon the vitality of the pericementum, and how could a hole bored in the bone with a drill be endowed with pericemental life? The idea was absurd, and we had prepared a gentle rebuke for the unscientific presumption of the California dentist. But before the publication of the review of his pamphlet, came letters from reliable Californian friends and correspondents, who avowed that they had seen this done, and with apparent success. In the September number we published one such, but with rather a feeling of compassion for the credulity manifested. This letter was followed by others from candid eastern men, who saw the operation performed during a visit to the Pacific coast last summer. An article was written for this journal by Dr. Julien W. Russell, of Brooklyn, who was one of these, but the whole affair seemed so unscientific that it was withheld, notwithstanding the candid manner in which the subject was presented. We waited for yet further testimony, for the assertion was now made that teeth which had been extracted for some time and which were in a desiccated condition, were thus implanted.

Finally, Dr. Younger came east for the avowed purpose of demonstrating this dental absurdity, and has visited New York, Philadelphia, Boston and Buffalo, in each place exemplifying the operation. We were bound in all courtesy to receive him and give him an opportunity to attempt the feat, for he asked no money of any one; he had no patent gim-cracks to sell, and his clinics were given freely, without pay, and seemingly for only the most commendable of purposes. He was contributing his time and money for what he believed was the good of dentistry, and notwithstanding the fact that we believed him to be mistaken in his theories and irrational in his practice, we were anxious to see what would be the effect of such an operation, and accordingly earnestly invited him to visit Buffalo.

We have been thus, perhaps, unnecessarily candid in reviewing the presentation of the new operation, lest we might be charged with a too ready credulity. Concerning the operations in New York, Philadelphia and Boston, we have no personal knowledge, but we are assured that they so far seem to be successful. It appears, also, to be established by sufficient testimony, that a patient was presented in New York for whom had been implanted a tooth in an artificial socket more than a year previously, and it was impossible to detect any marked differences between it and its natural neighbors.

Of the clinic in Buffalo, given in our own office, upon a patient of our own selection, and in the presence of Dr. Park, professor of surgery in the University of Buffalo, and that of a number of our city dentists, we can speak knowingly. Dr. Younger labored under disadvantages, for, not knowing what was wanted, nothing had previously been prepared for him. The patient was a young man, a student in the medical college. Nearly a year before he had lost a right inferior second bicuspid, and it was proposed to insert a tooth in its place. Not a trace of the socket remained, and there was considerable absorption of the alveolus, which made necessary a tooth with a long crown. None such could be readily found, until Dr. Younger himself visited the office of one who extracts a great many teeth, when, out of a bushel or more of dried up, long extracted dental organs, he found one which the donor said had rested in the dental golgotha for at least three years, among thousands of its foul companions. This was carefully prepared by removing the debris from the pulp chamber and canal, thoroughly disinfecting it with a one to one thousand solution of bichloride of mercury, and filling the root with gutta-percha. It was then left in a warm solution of the corrosive sublimate while the socket was prepared.

This was accomplished by carefully dissecting back the gum, and with a small trephine boring a hole in the alveolus, which was enlarged to the proper size and shape with burs. The tooth was occasionally tried in the socket to determine the proper direction, size and depth, and when these were secured it was forced down to its proper position and left there, the socket having been previously thoroughly syringed out to remove all debris. Its perfect occlusion was now secured by dressing down the cusps a little, when the case was dismissed. So accurate was the fit that the tooth was held firmly in position by mechanical adaptation alone, and no ligatures or other appliances were used to retain it. The occlusion with the superior tooth was, of course, sufficient to prevent its rising in the socket. No after-treatment was recommended, but the whole thing was left to the care of nature. The operation was dexterously performed, the occlusion was perfect, and the pain experienced by the patient was not greater than that necessary in filling a cavity in a rather sensitive living tooth.

At this present writing the tooth has been in position about a week, and aside from a very little soreness, the necessary result of the wound, there has been no bad symptoms, and the case appears to be progressing favorably. Of course the time is not sufficient to afford any reliable index of what the final result will be, and had we not credible testimony that cases analogous to this have gone on to apparent success for a year and more, it would not be quoted. As, however, the operation is attracting some attention in the profession, we have thought it not out of place to detail the manner of its performance, reserving the right to report further upon this instance at a subsequent day.

If this operation should prove to be a feasible and successful one, it will certainly require the revision of our ideas of physiological law. It must not, however, be understood that we are committed to it, or are fully convinced that it is a practice which may unhesitatingly be commended. We are content to wait further developments. And yet, if a smooth ivory peg may be driven into bone and in time be encapsuled within the tissue, or perhaps united to the circumjacent bone, why is it not possible for a tooth to be made to grow in an artificial socket? We know that the spongy portions of the maxillæ are the most accommodating of the bodily tissues, and will submit to almost any surgical or traumatic interference and yet return to a state of health. We also know that the proliferation of bone corpuscles is not entirely dependent upon periosteum, but that cavities artifically made in bones will be healed from the bottom by granulation. In numerous instances we have replanted teeth that have been out for many hours, and some of these have remained in good condition for ten years, and are seemingly perfect yet. At the present time there is under our care the case of a boy of eight years of age, whose central incisor was knocked out, and practically remained out, for more than forty hours. The tooth was incomplete in growth, and there was nothing like a finished foramen, the end being open the full size of the root. Yet this was inserted, after proper preparation, and now, three weeks and more after the accident, it appears to be well united and comparatively solid and healthy. But all this is but little analogous to the implantation of foreign teeth in artificial sockets.

Dr. Younger is careful to select teeth the roots of which are apparently covered with pericementum, no matter in what a desiccated condition this may be. He believes in the persistent vitality of this membrane. Although we are aware that membraneous tissue differs from most of the other tissues of the body in the readiness with which its presence, even when long dead, is tolerated, as is evidenced by its occasional use in surgery, we cannot believe that it is because it has retained any vitality. This seems to us absurd. That Dr. Younger has made his only failures, according to his own statement, in the insertion of teeth that were without the dried pericemental membrane, may be due to the fact that it may act as a protection to the primary deposition of protoplasmic matter, which finds a resting place in its meshes, and thus acts like the cells of the sponge in artificial grafting.

It would be interesting to determine just what is the nature of the relation of the tooth and bone in these implanted cases—whether a pericemental membrane is formed and the organ held in position in the usual way, or whether there is a solid, bony union. That it is not a merely mechanical adhesion seems abundantly proved, for in some of Dr. Younger's cases the gum has been found perfectly adherent to the neck of the tooth. It is to be hoped that, at some time, some of those implanted teeth that seem entirely healthy may be extracted and critically examined, for we think it must be admitted that a union of some kind has really been established between teeth long extracted and the walls of artificially formed sockets.

